# Length–Weight Distribution of Non-Zero Elements in Randomized Bit Sequences

**DOI:** 10.3390/s25123825

**Published:** 2025-06-19

**Authors:** Christoph Lange, Andreas Ahrens, Yadu Krishnan Krishnakumar, Olaf Grote

**Affiliations:** 1 School of Engineering—Energy and Information, Hochschule für Technik und Wirtschaft Berlin, University of Applied Sciences, 10313 Berlin, Germany; 2 Faculty of Engineering, Hochschule Wismar, University of Applied Sciences: Technology, Business and Design, 23966 Wismar, Germany; andreas.ahrens@hs-wismar.de (A.A.); yadu.krishnakumar@hs-wismar.de (Y.K.K.); 3 Escuela Técnica Superior de Ingeniería y Sistemas de Telecomunicación (ETSIST), Universidad Politécnica de Madrid, Campus Sur, Calle Nikola Tesla s/n, 28031 Madrid, Spain; olaf.grote@alumnos.upm.es

**Keywords:** randomized bit sequences, burst, test, probability, gap process, gap distribution

## Abstract

Randomness plays an important role in data communication as well as in cybersecurity. In the simulation of communication systems, randomized bit sequences are often used to model a digital source information stream. Cryptographic outputs should look more random than deterministic in order to provide an attacker with as little information as possible. Therefore, the investigation of randomness, especially in cybersecurity, has attracted a lot of attention and research activities. Common tests regarding randomness are hypothesis-based and focus on analyzing the distribution and independence of zero and non-zero elements in a given random sequence. In this work, a novel approach grounded in a gap-based burst analysis is presented and analyzed. Such approaches have been successfully implemented, e.g., in data communication systems and data networks. The focus of the current work is on detecting deviations from the ideal gap-density function describing randomized bit sequences. For testing and verification purposes, the well-researched post-quantum cryptographic CRYSTALS suite, including its Kyber and Dilithium schemes, is utilized. The proposed technique allows for quickly verifying the level of randomness in given cryptographic outputs. The results for different sequence-generation techniques are presented, thus validating the approach. The results show that key-encapsulation and key-exchange algorithms, such as CRYSTALS-Kyber, achieve a lower level of randomness compared to digital signature algorithms, such as CRYSTALS-Dilithium.

## 1. Introduction

When generating random numbers on personal computers, they are, strictly speaking, *pseudorandom,* as they are usually produced using deterministic numerical algorithms [1]. Good pseudorandom sequences show a behavior that is very similar to that of real random sequences, which are usually more complex to generate, e.g., by using physical noise sources or mechanical devices. Hence, it is important to test computer-generated pseudorandom sequences—called random sequences throughout this article, as is common in the literature—for whether their elements are independent of each other and whether they show a desired probability distribution.

Randomized bit sequences consisting of a set of zero and non-zero elements are of particular interest. In communications, they are often used to simulate random information-bearing bitstreams, while in cryptography, they form the basis of many ciphering algorithms. The sequence of zero and non-zero elements, namely zeros and ones, is regarded as a set of random variables. These variables should show an identical distribution—a discrete uniform distribution in the considered case—and they should be independent of each other.

This leads to a fundamental concept in probability theory and statistics: the IID (independent and identically distributed) condition, which describes a scenario where a set of random variables meets the two mentioned key criteria—independence and identical distribution [2,3]. Each element in a random variable, which means a sequence of non-zero and zero elements in the considered case, follows the same probability distribution and is not influenced by any other element of the sequence. Furthermore, the sequence is balanced, i.e., it has the same number of zero and non-zero elements. This is the special case of a discrete uniform distribution of the zero and non-zero elements. Throughout this article, this condition will be denoted as the *IID-DU* condition, meaning that each of the IID random variables shows a discrete uniform (DU) distribution.

This article presents a gap-based method that complements traditional test suites by directly evaluating whether a binary sequence demonstrates independent and identically distributed behavior and follows a discrete uniform distribution. By analyzing the intervals—or gaps—between significant bits, the method uncovers structural deviations that may be missed by frequency-based tests. It offers a promising addition to established test suites such as the NIST frequency test, TestU01, and DieHard [4].

There are several tests that can check empirical random data, with regard to the question of what distribution they show, and others that test with regard to the problem of whether they are independent [5,6]. Independence is often tested via autocorrelation; for example, the *Durbin–Watson* test, which is a hypothesis test [7]. Other test strategies follow graphical methods, such as plotting the data over time or analyzing them through scatter plots [8]. The distribution of the elements can be analyzed through histograms or density plots, where the distribution of different subsets of the data is studied to determine whether they originate from the same distribution. Significant differences in means or variances across data subsets may indicate that they are not identically distributed. Furthermore, tests such as the *Kolmogorov–Smirnov* test [9] or the *Anderson–Darling* test [10,11] can be used to test which probability distribution the observed data set obeys through a hypothesis test.

For the purpose of testing whether a binary sequence shows a discrete uniform distribution of the two elements, typical tests for random numbers include the *frequency test*, the *runs test* [12], and the *gap test* [4].

In this article, tests regarding the IID-DU property of binary random sequences are extended with the help of the gap distribution between the non-zero elements in a random sequence, whose importance for the IID-DU assumption has so far been insufficiently considered. The novelty of this work lies in the analysis of burst structures within the studied random sequences and the length and weight of the non-zero elements therein. It provides an alternative way to test whether the IID-DU condition has been met. In this work, a new method for testing the randomness of random bit sequences is presented. It is based on the application of *gap processes*, which have been successfully applied in different fields of communication engineering, such as modeling short-wave radio transmission channels [13,14], describing and analyzing TCP/IP-based data traffic (Transmission Control Protocol/Internet Protocol) [15,16], and analyzing the randomness of bit sequences [17,18].

Several suitable probability distributions are considered, and their parameters are derived for empirical random sequences generated using different approaches. Using an error criterion, the best-fitting distribution among the considered models is identified, enabling an assessment of whether the IID-DU condition has been satisfied. This is important for the practical application of randomized bit sequences in the fields of communication and cryptography, as perfect IID-DU conditions are rare in real-world data.

Furthermore, randomness plays a crucial role in many cryptographic systems, for which two key properties of a secure cipher have been identified: confusion and diffusion [19]. Gap-based burst analysis effectively evaluates the randomness of cryptographic bit sequences, including those from post-quantum algorithms under NIST standardization, by highlighting the deviations from ideal randomness. This method can detect early signs of inadequate independence or other anomalies that may indicate structural weaknesses in an encryption system. Confusion aims to eliminate any correlation between plaintext and ciphertext through non-linear transformations, such as S-boxes (substitution boxes). S-boxes substitute blocks of bits with other bit blocks in a non-linear fashion to introduce confusion [20]. By contrast, diffusion ensures that the statistical properties of the plaintext are spread across a large area of the ciphertext, often achieved through P-boxes (permutation boxes). P-boxes reorder the positions of bits to achieve diffusion [20]. Both are essential components of block cipher algorithms, including the Advanced Encryption Standard (AES).

The remainder of this article is organized as follows. In Section 2, the modeling basics are introduced, and exemplary parameters and functions are given. In Section 3, exemplary sequences are analyzed, and the results are presented and compared with theoretical findings. In Section 4, several methods for generating suitable random sequences are analyzed, their advantages and disadvantages are discussed, and exemplary results are presented. In Section 5, post-quantum cryptographic approaches are discussed, and related exemplary results are presented. Finally, Section 6 provides concluding remarks, while Section 7 outlines future work and provides an outlook.

## 2. Model Basics

### 2.1. General Probability Description

The characteristics of random bit sequences can be described by the gaps between the non-zero elements (so-called *gaps*) or by the gaps between the zero elements (so-called *blocks* [21]) in the random stream of non-zero and zero elements, i.e., “1” and “0”.

**Figure 1:**
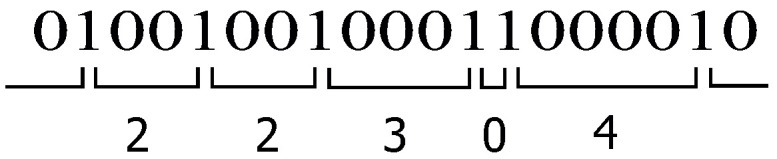
Modeling the intervals between non-zero elements by gaps (a non-zero element, represented by “1”, is separated by gaps of different lengths, represented by “0”, describing zero elements).

In this article, the gaps between the non-zero elements are used for description and analysis. Figure 1 shows an exemplary snippet of a random sequence consisting of zero elements “0” and non-zero elements “1”, with the associated gap length written beneath, i.e., the number of zero elements between successive non-zero elements.

Due to the random nature of the phenomena under consideration, the mathematical treatment requires elements of probability theory. The necessary tools are introduced in the following.

The *gap-distribution function*

(1) u(k)=P(Y≥k),k=1,2,3,…

denotes the probability that a gap *Y* is greater than or at least equal to a given number *k* (with k∈N). By this definition, u(k) represents a kind of complementary cumulative distribution function, since it is useful in the topic under investigation to study the question of how often the random variable (gap *Y*) is on or above a particular level *k* [13]. However, the related *gap-density function*

(2) v(k)=P(Y=k),k=1,2,3,…,

describes the probability that a gap *Y* of length *k* is observed [3,22].

The interrelationship between the gap distribution and gap-density function is given by

$$\begin{array}{cc} u(k)= & \;\;v(k)+v(k+1)+v(k+2)+\cdots \\ u(k+1)= & \;\;v(k+1)+v(k+2)+\cdots \end{array}$$

and results in

(3) v(k)=u(k)−u(k+1).

The value v(k) is thus the difference of the values u(k) and u(k+1), which corresponds with the theory of discrete random variables. The gap process can be understood as a sequence of intervals of different lengths (see Figure 1), and therefore the gap distribution or the gap density ensures a sufficient description of the underlying random sequence, respectively. The model characteristic comprises the independence of the gaps themselves. Investigations in the 1960s and 1970s have shown that this assumption is not always guaranteed, but can be seen as a good practical approximation [23].

The gap-density function v(k) provides information on the probability of gaps of certain lengths. Therefore, v(k) exhibits a close relationship to the run-length analysis—as part of the National Institute of Standards and Technology (NIST) tests, which search for uninterrupted sequences of identical elements (bits) [4]. The gap-density function contains information regarding all runs of identical zero elements within the random sequence.

### 2.2. Probability Distributions

Some popular distributions for describing random gap processes are introduced and are used exemplarily throughout this article to obtain numerical results. However, the method focuses on discrete distributions, as it analyzes inherently discrete events—specifically, the occurrences of 1-elements (or bit errors) within a bitstream—without involving continuous-time signal sampling. By concentrating on discrete gap lengths, the approach facilitates direct comparison with established two-parameter models, such as the Weibull and Wilhelm distributions, which have demonstrated effectiveness in analogous contexts (e.g., inter-packet arrival times or bit error gaps). This discrete emphasis also simplifies the evaluation of deviations using squared error metrics, ensuring that the analysis remains appropriately aligned with the discrete nature of burst-like phenomena [24].

Table 1 provides the characteristic probability distribution functions and probability density functions of the exponential, Weibull, and Rayleigh distributions (see, e.g., [15,25]). Here, it is worth noting that for $\alpha_{w}=1$, the Weibull distribution function equals the exponential distribution. This function has been widely accepted in the research community when analyzing gap processes, e.g., when considering the gaps between successive TCP packets in data networks (e.g., [15,26]) or when analyzing the distribution of bit errors in wireless communications [14].

For independent non-zero elements (also known as memoryless or non-bursty elements), the ideal gap-distribution function results in

(4) $$u\left(k\right)={\left(1-p_{e}\right)}^{k}\;0\leq k<\infty$$

with the parameter $p_{e}$ defining the probability that a given element in a sequence is non-zero [18]. With (3), the gap-density function v(k) is obtained as

(5) $$v\left(k\right)={\left(1-p_{e}\right)}^{k}\cdot p_{e}\;.$$

The parameter $p_{e}$ describes the probability that an arbitrary value of the random sequence adopts the value 1. For random bit sequences with a uniform distribution of “0” and “1”, $p_{e}=0.5$ is assumed. When a “1” is considered a bit occurrence, this parameter is referred to as the *bit occurrence probability* (BOP) [18].

**Figure 2:**
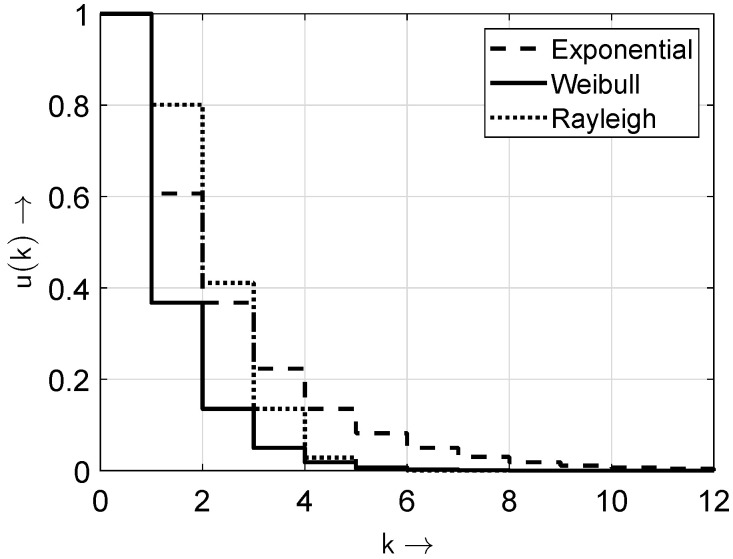
Probability distribution functions of the distributions from Table 1.

**Figure 3:**
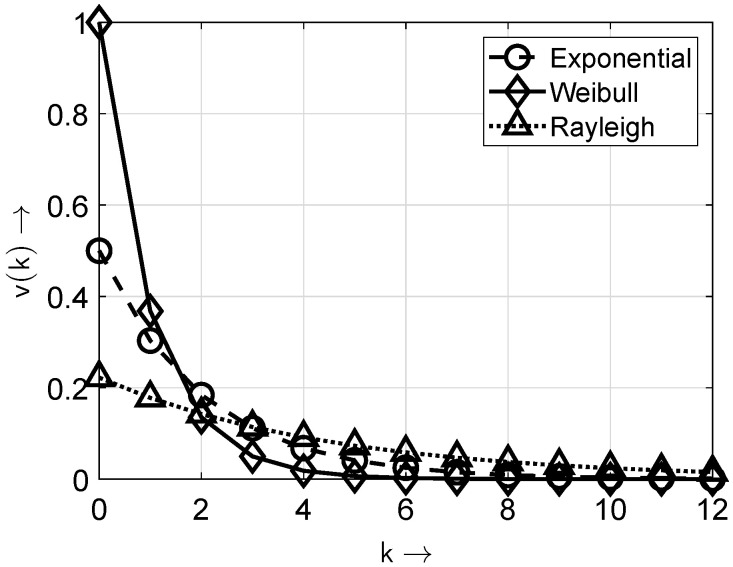
Probability density functions of the distributions from Table 1. Note: only the values of v(k) at integer *k* values are non-zero; the connecting lines are included solely for improved appearance and discriminability.

In Figure 2 and Figure 3, the distributions and probability density functions of the distributions given in Table 1 are depicted for the exemplary parameters $\beta_{e}=1/2$, $\alpha_{w}=1$, $\beta_{w}=1$, and $\beta_{r}=2/3$.

Probability distributions with two parameters, e.g., the Weibull and Rayleigh distributions, are especially suitable when non-zero elements are highly concentrated in the random bit sequence. These are also known as models with memory. In such cases, successive elements of the random sequence are not independent of each other. Only cases with no memory (memoryless models)—where a subsequent non-zero element does not depend on a preceding non-zero element—can be sufficiently described by a distribution with a single parameter (e.g., an exponential distribution).

For this reason, the exponential distribution is a favored distribution function for gaps. Thus, throughout this article, a random sequence with an exponential gap distribution is referred to as an *ideal sequence*.

This is particularly important for the frequency test. Even with $p_{e}=0.5$, the independence of the non-zero elements cannot be guaranteed automatically, as v(0)=0.5 is not automatically fulfilled.

A strict threshold for v(0) is impractical, as randomness exists on a spectrum rather than as a binary trait. While v(0)=0.5 reflects the ideal for independent, uniformly distributed sequences, slight deviations can occur due to benign factors like sampling noise. Conversely, even sequences with v(0)=0.5 may conceal structural dependencies. Relying solely on a fixed cutoff risks both false rejections and false acceptances. Therefore, a more nuanced approach—evaluating the nature and extent of deviations from 0.5—offers a better balance of flexibility and rigor in detecting non-randomness.

Via the analysis of bit error distributions for short-wave radio transmissions, the approach

(6) $$u\left(k\right)=\left[{\left(k+1\right)}^{\alpha }-k^{\alpha }\right]\cdot e^{-\beta \cdot k}\;for\;k=0,1,2,3,\ldots \;,$$

was practically found and verified for characterizing random sequences using gap processes [13]. It is known as the *Wilhelm distribution*. Thus, the auxiliary condition holds

(7) $$\lim_{k\to \infty }e^{-\beta \cdot k}=0\;\mathrm{for}\;\beta >0\;\mathrm{with}\;p_{e}=\beta^{\alpha }$$

Here, infinite gap lengths *k* are allowed. In a simulation, the gaps have to be appropriately limited. It is worth noting that for α=1, the Wilhelm distribution function equals the exponential distribution given in Table 1.

**Figure 4:**
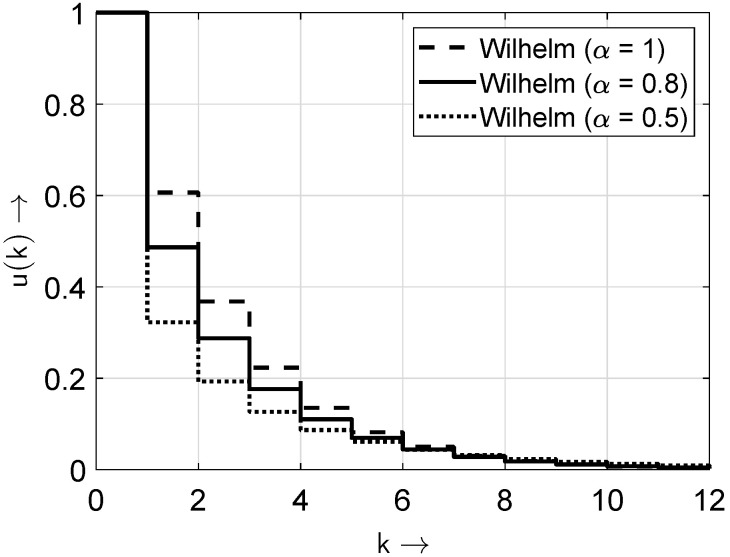
Probability distribution functions of the Wilhelm distribution for different values of the parameter α.

**Figure 5:**
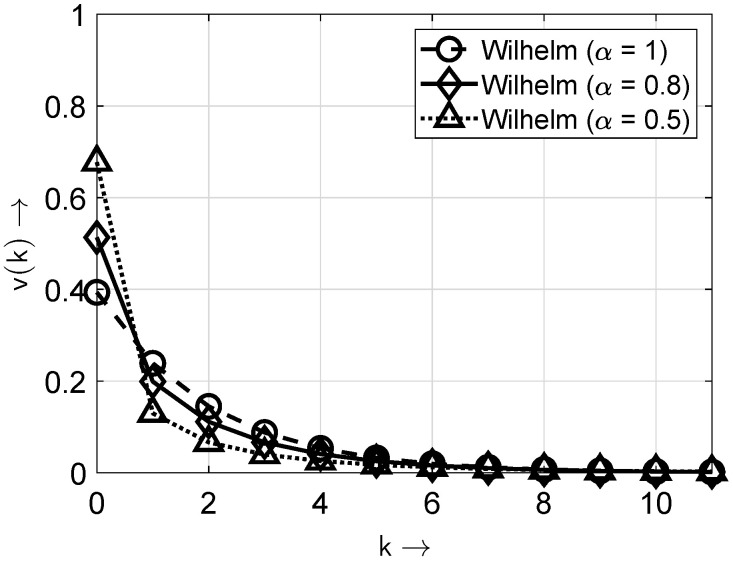
Probability density functions of the Wilhelm distribution for different values of the parameter α. Note: only the values of v(k) at integer *k* values are non-zero; the connecting lines are included solely for improved appearance and discriminability.

In Figure 4 and Figure 5, the distribution and probability density functions of the *Wilhelm* distribution according to (6) are shown for the exemplary parameter $p_{e}=1/2$ and $\beta =p_{e}^{\left(1/\alpha \right)}$ and for different values of the parameter α.

The parameter (1−α) indicates how concentrated the bits of the random sequence are. Therefore, it represents a kind of concentration or burstiness factor. The desired values are (1−α)=0 or α=1. Then, the considered channel (or model in general) is memoryless.

For α=1, the relationship $\beta =p_{e}^{\left(1/\alpha \right)}=p_{e}$ holds, and (6) can be directly rewritten as

(8) $$u\left(k\right)=e^{-p_{e}\cdot k}\;for\;k=0,1,2,3,\ldots \;.$$

The Taylor series expansion of the exponential function $e^{-\;x}$ for small *x* provides an option for rewriting the function $e^{-\;x}$ (with [27]) as

(9) $$e^{-\;x}=1-x+\frac{x^{2}}{2}-\frac{x^{3}}{6}+\frac{x^{4}}{24}+\cdots \;.$$

A suitable termination of the series leads to the approximation

(10) $$e^{-\;x}\approx 1-x$$

for small *x*. Then, the gap-distribution function results in

(11) $$u\left(k\right)={\left(1-p_{e}\right)}^{k}\;for\;k=0,1,2,3,\ldots \;.$$

This result is known from probability theory (see (4)). According to Wilhelm (8), the approach in this case corresponds with the exponential distribution (see Table 1).

**Figure 6:**
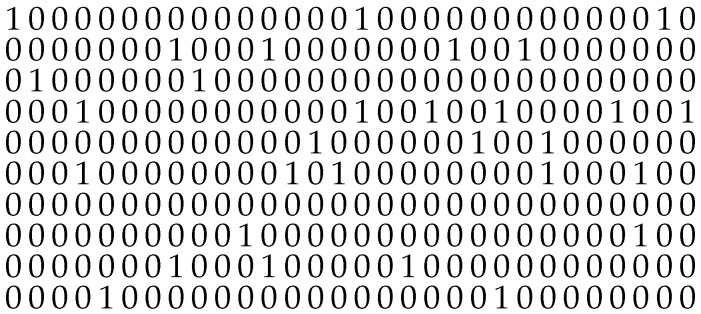
Exemplary distribution of non-zero elements (denoted as “1”) and zero elements (denoted as “0”) within a binary sequence in a non-bursty scenario, illustrating independently distributed non-zero elements.

**Figure 7:**
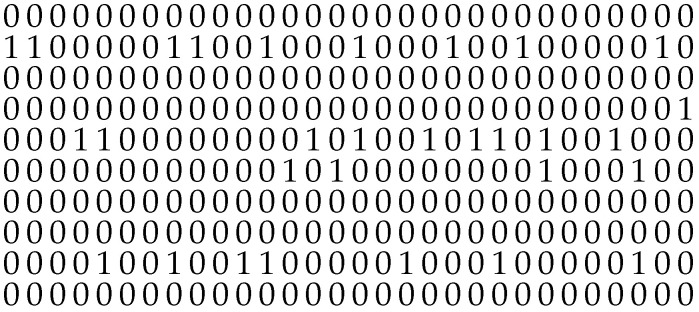
Exemplary distribution of non-zero elements, represented by “1”, and zero elements, represented by “0”, within a binary sequence exhibiting a bursty appearance of the non-zero elements.

It is worth noting that for values (1−α)>0 or α<1, the non-zero elements appear concentrated. Here, the gap-density function v(k) shows higher values for short gap lengths and lower probabilities for longer gap lengths. Such scenarios can be described by distributions with two or more parameters, e.g., the Wilhelm distribution or the Weibull distribution. Then, the investigated sequences show a non-IID-DU behavior. Figure 6 and Figure 7 illustrate the differences in the bit sequences with and without bursty behavior. For illustration purposes, the BOP is set to $p_{e}=0.1$.

Knowing the gap-density function v(k), the averaged gap length can be calculated as the expectation of a discrete random variable [3]

(12) $$E\left(k\right)=\sum_{k=0}^{\infty }k\cdot v\left(k\right)\;.$$

Expanding (12) leads to

(13) $$E\left(k\right)=\sum_{k=0}^{\infty }k\cdot v\left(k\right)=0\;v\left(0\right)+1\;v\left(1\right)+2\;v\left(2\right)+\ldots$$

and with v(k)=u(k)−u(k+1) (see (3)), the sum can be rewritten as

(14) $$\sum_{k=0}^{\infty }k\cdot v\left(k\right)=1\;\left(u\left(1\right)-u\left(2\right)\right)+2\;\left(u\left(2\right)-u\left(3\right)\right)+\ldots$$

resulting in

(15) $$\sum_{k=0}^{\infty }k\cdot v\left(k\right)=u\left(1\right)+u\left(2\right)+\ldots \;.$$

Now, u(0) is added and subtracted from the right-hand side of the equation, and the result is

(16) $$\sum_{k=0}^{\infty }k\cdot v\left(k\right)=-u\left(0\right)+u\left(0\right)+u\left(1\right)+u\left(2\right)+\ldots \;.$$

Finally, the expression

(17) $$\sum_{k=0}^{\infty }k\cdot v\left(k\right)=\sum_{k=0}^{\infty }u\left(k\right)-u\left(0\right)$$

is obtained for u(0)=1, resulting in

(18) $$E\left(k\right)=\sum_{k=0}^{\infty }k\cdot v\left(k\right)=\sum_{k=0}^{\infty }u\left(k\right)-1.$$

This fulfills the probability for the BOP $p_{e}$ in the following way:

(19) $$E\left(k\right)+1=\frac{1}{p_{e}}$$

with E{·} denoting the expectation functional.

Then,

(20) $$\sum_{k=0}^{\infty }u\left(k\right)=\frac{1}{p_{e}}$$

is valid. Equation (4) can be interpreted as an alternative criterion for testing whether the IID-DU condition is met. In the case of $p_{e}=0.5$ and $v\left(0\right)=p_{e}$, it is fulfilled; otherwise, it is not. To prove this, according to (4), for the ideal gap-distribution function u(k), the approach becomes

(21) $$\sum_{k=0}^{\infty }u\left(k\right)=\sum_{k=0}^{\infty }{\left(1-p_{e}\right)}^{k}.$$

By applying the geometric series to $|(1-p_{e})|<1$, the result

(22) $$\sum_{k=0}^{\infty }u\left(k\right)=\frac{1}{1-(1-p_{e})}=\frac{1}{p_{e}},$$

already known from (20), is obtained. In fact, various distribution functions can be used here, but only the exponential distribution fulfills the condition v(0)=0.5.

It should be noted that the value $p_{e}=0.5$ is relatively high with regard to the requirement to be “small” when applying the geometric series for approximation purposes. This is reflected in the numerical results obtained in later parts of this article.

### 2.3. Uniform Distribution of the Sequence’s Elements and Testing

The IID-DU requirement presumes that the condition $v\left(0\right)=p_{e}$ is fulfilled with $p_{e}=0.5$. This implies that, following a non-zero element, the probability of another non-zero element occurring immediately is 0.5 (or 50%). Furthermore, a balanced sequence is required—one in which the number of zero and non-zero elements differs by no more than a single element.

Initially, even in cases where these requirements are fulfilled, this gives no information about the distribution of the gaps between the non-zero elements. The gap distribution follows (22), which can be obtained via different distribution functions. It can indeed be fulfilled by using different distribution functions; however, the condition $v\left(0\right)=p_{e}$ may be violated.

The value v(0) indicates the probability that after a non-zero element in the distance k=0, a non-zero element immediately follows again. This probability is set to 0.5 or 50%, based on the assumption that the zero elements (“0”) and non-zero elements (“1”) show a discrete uniform distribution (two-point or *Bernoulli* distribution, [28]) within the random sequence.

Starting from (4) with (3) for v(0), the following result is obtained:

(23) $$v\left(0\right)=u\left(0\right)-u\left(1\right)=1-u\left(1\right)=1-\left(1-p_{e}\right)=p_{e}$$

Assuming independent gaps in the considered random sequence (i.e., there are no ties between the elements or bits), v(0)=0.5 holds. The probability that after a non-zero element, the next element is also a non-zero element is 50% or 0.5.

In order to verify whether the sequences show the desired properties of a uniform distribution of zero and non-zero elements, tests are necessary. In particular, the *frequency test* and the *run-length test* [4] can be used.

In an *n*-bit sequence, the Hamming weight [29,30,31] should be approximately n/2 in cases where the number of non-zero elements (“1”) is equal to the number of zero elements (“0”). Then, the sequence is said to be balanced.

The frequency test measures the difference between the number of ones and zeros in an *n*-bit sequence. If it is determined to be nearly the same, the zero and non-zero elements are uniformly distributed throughout the sequence.

The sequence’s elements adhere to a discrete uniform distribution expressed by the condition v(0)=0.5. Conversely, it may well be that there are 50% zeros and 50% ones in the sequence, but the condition v(0)=0.5 is not fulfilled. In this case, the non-zero elements occur in a concentrated manner with a certain clustering, i.e., in bursts (see the Wilhelm distribution for values α<1). Thus, the determination of the frequency of non-zero elements, or the Hamming weight, does not determine whether the non-zero elements are concentrated and thus whether the IID-DU condition is fulfilled.

The runs test [12] is based on determining the number of successive identical elements in a sequence; this is called a *run*. A run is defined as an uninterrupted sequence of identical elements (zeros or ones in the considered case) in the sequence. By using the runs test, the number of such runs in a given sequence is counted; if the sequence is random, there should not be too many, but also not too few, runs (very few runs point toward a bursty characteristic; very many runs indicate oscillatory behavior).

It is worth noting that the theory behind the runs is closely related to the gap-density function. For an ideal sequence, where the gaps are exponentially distributed with $p_{e}=0.5$, at least half of the total number of runs of zero elements or non-zero elements should have length zero, at least one-fourth should have length one, at least one-eighth should have length two, and so on. This can be calculated directly from (5) with $p_{e}=0.5$.

It should be noted that the gap-density function v(k) and the corresponding parameter v(0) with $v\left(0\right)=p_{e}=0.5$, according to the IID-DU condition, serve as an indicator for randomness if the underlying density function contains all gap lengths rather than a few distinct lines within the density function. An example of the latter case would be the sequence …110011001100…. This sequence can be regarded as balanced with $p_{e}=0.5$, but the corresponding gap-distribution function contains only a few gap lengths. The density function has two distinct lines with v(0)=0.5 and v(2)=0.5. However, as the gap-density function has degenerated to two distinct lines, the underlying bit sequence should be classified as non-random. In this work, we focus on detecting deviations from the ideal density function v(k), defined in (5). Density functions with only a few distinct lines require separate analysis and will lead to a non-randomized behavior with high probability.

Determining the gap-density function necessitates a substantial sample size to identify potential violations of independence, especially when gaps occur in bursts, which increases the frequency of shorter gaps. To reliably capture the distribution of longer gaps, sequence lengths between 10,000 and 20,000 bits are recommended.

## 3. Burst Analysis Within Random Bit Sequences

### 3.1. Theoretical Concept

**Figure 8:**
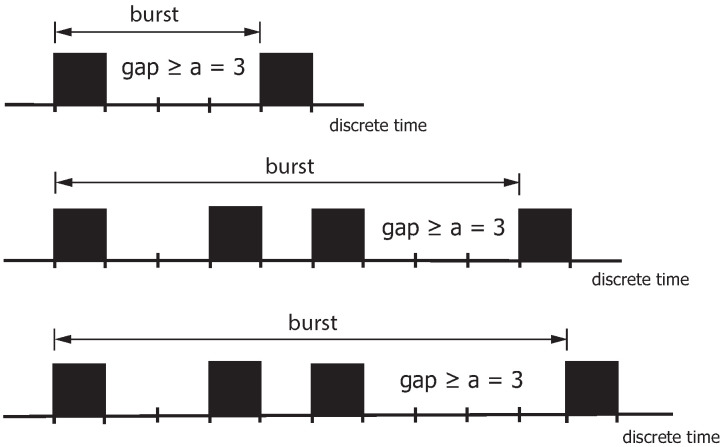
Definition of bursts with the exemplary distance parameter a=3.

When analyzing binary sequences by gaps, a burst is based on the distribution of the zero and non-zero elements within the binary sequence and is defined as a pattern that begins with a non-zero element and ends with the next non-zero element when at least *a* zero elements are between them. The burst terminates with the last non-zero element. This non-zero element is the starting point of the next burst. The parameter *a* is also called the distance parameter (gap) between two non-zero elements. If the gap after a non-zero element is greater than or equal to the distance parameter (gap) *a*, the burst is regarded as terminated. Figure 8 highlights the burst definition with a=3. The burst ends when the gap after a non-zero element is greater than or equal to the distance parameter *a* (in the example, a=3). The proportion of these gaps is characterized by the parameter u(a), referred to as the gap-distribution function u(k). This definition states that a burst can contain more than one non-zero element.

When analyzing such patterns, the weights—defined as the number of non-zero or “1” elements—and the lengths of the sequences play a critical role.

**Figure 9:**
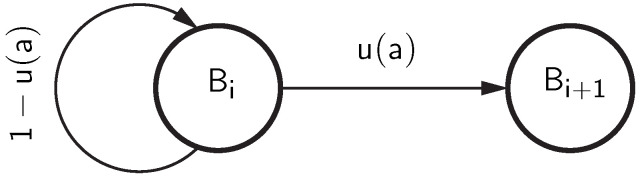
Non-zero distribution using a Markov chain.

Figure 9 illustrates the generation of bursts. The Markov chain [32] is started from state $B_{i}$, meaning that the burst is assumed to have already begun with a non-zero element. The Markov chain remains in state $B_{i}$ as long as the next occurring non-zero element—belonging to burst *i*—has a gap to the previous non-zero element that is shorter than *a*. When moving to the next non-zero element, the burst will be finished when the gap *k* is greater than or equal to *a*, i.e., k≥a. The Markov chain is then in state $B_{i+1}$, indicating that the next burst has started.

The number of bursts $z_{B}\left(a\right)$ with the distance parameter *a* in a given sequence with $z_{f}$ non-zero elements results in

(24) $$z_{B}\left(a\right)=z_{f}\cdot u\left(a\right)\;.$$

Each non-zero element can be a burst start. The burst ends when the gap *k* to the following non-zero element is greater than or equal to the distance parameter *a*. The associated probability is u(a), When $z_{f}$ non-zero elements populate the random sequence, only $z_{f}\cdot u\left(a\right)$ bursts can occur. With the distance parameter a=0 and u(0)=1, every non-zero element represents a burst’s starting point. Then, $z_{B}\left(a\right)=z_{f}$ holds.

In (24), the average number of non-zero elements *g* within a burst is calculated as

(25) $$E\left(g\right)=\frac{z_{f}}{z_{B}\left(a\right)}=\frac{1}{u(a)}\;.$$

This value provides information about how strongly the non-zero elements are concentrated in the bursts, which is especially interesting for sequences where the IID-DU assumption is violated.

The number of non-zero elements per burst can be calculated by the weight distribution P(g) as a function of the distance parameter *a*, resulting in

$$\begin{array}{cc} P(g=1)= & \;\;u(a)\\ P(g=2)= & \;\;u(a)\cdot [1-u(a)]\\ \vdots & \;\;\\ P(g)= & \;\;u\left(a\right)\cdot {\left[1-u\left(a\right)\right]}^{g-1}\;. \end{array}$$

**Figure 10:**
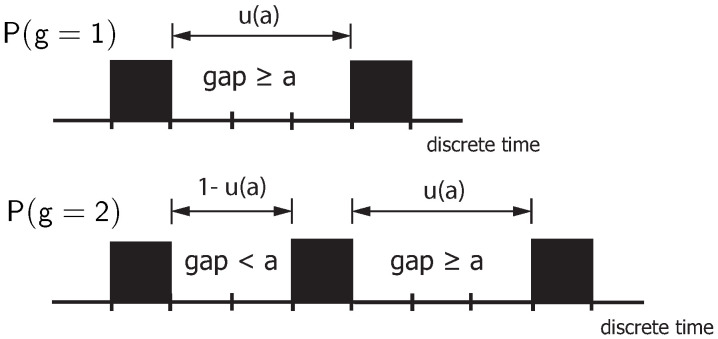
On the determination of the burst-related weight distribution P(g): Influence of the distance parameter *a* for different weights *g*.

The explanation makes use of Figure 10. A burst starts with a non-zero element and ends when the gap toward the following non-zero element is greater than or equal to the distance parameter *a*. Thus, the burst has weight g=1 if, at the beginning of the burst, the gap is equal to or greater than *a*. The probability of such an event is given by u(a). The burst has weight g=2 if, at the beginning of the burst, a gap smaller than *a* initially occurs (with probability (1−u(a))), followed by a gap greater than or equal to *a*.

**Figure 11:**
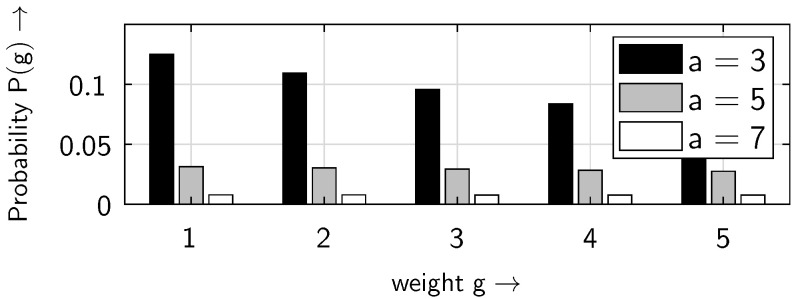
Weight distribution of the non-zero elements P(g) within the bursts for different values of the distance parameter *a*, at a BOP of $p_{e}=0.5$, assuming the ideal gap distribution defined in (4), for small weights *g*.

**Figure 12:**
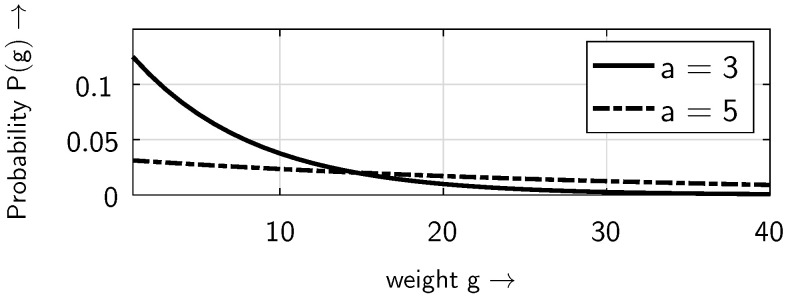
Distribution of the non-zero elements P(g) within the bursts for different values of the distance parameter *a*, at a BOP of $p_{e}=0.5$, assuming the ideal gap distribution defined in (4), for larger weights *g*.

Figure 11 and Figure 12 show the distribution of the non-zero elements within the bursts. For small weights *g*, the probability P(g) decreases as the distance parameter *a* increases (Figure 11). However, this behavior is reversed for larger weights *g* (Figure 12). This is because, as the distance parameter *a* increases, the number of bursts decreases, i.e., the number of non-zero elements per burst rises. It should be noted that in Figure 12—as in Figure 11—there are only discrete weight values of *g* on the horizontal axis; the continuous line is included to make the behavior, in particular the crossover point, more visible.

### 3.2. Verification

Assuming that the non-zero elements of the sequence are independently distributed (also referred to as non-bursty non-zero elements), the gap-distribution function u(k) can be derived as a function of the BOP $p_{e}$ [3]. With the BOP $p_{e}$, the probability that a single element in the sequence is zero is given by $\left(1-p_{e}\right)$. Therefore, the probability u(k)=P(Y≥k) that ≥k neighboring elements in the data stream are zero is given in (4).

The following sections illustrate the various approaches using a representative example. For this, the gap-distribution function (4) for independent non-zero elements indicates the starting point. The associated bit sequence is generated using the inversion transform method [1,18]. The exemplary bit sequence has a length of N=10,000 bits, containing $z_{f}=5042$ non-zero elements (corresponding to the sequence’s Hamming weight). This leads to $p_{e}=0.5$; thus, the sequence is nearly balanced.

Table 2 shows the number of measured bursts (compared with theory) for different values of *a*. It can be seen that with the increase in the distance parameter *a*, the number of bursts decreases; therefore, the number of non-zero elements per burst increases (see Table 2).

The simulation results align well with the theoretical values.

Furthermore, the weight distribution—defined as the number of non-zero elements per burst—was analyzed. Table 3 presents the simulated probabilities for different weights *g*.

The theoretical values, as derived from (4), are shown in Table 4.

The results demonstrate good agreement between the simulated and theoretical values.

In addition, the relative frequencies for the bursts’ length–weight distribution were analyzed, and the results are shown in Table 5. When evaluating bursts with a specific weight, e.g., g=1, the (absolute) frequencies shown in Table 3 appear. If all weights are considered, the number of bursts $z_{B}\left(a\right)$, as shown in the last row of Table 3, is obtained. Summing up yields $z_{B}\left(a\right)=624$ results in the considered example (see Table 5, last row, last column). Furthermore, Table 5 provides information about the bursts’ length distribution (see the last row). When all burst lengths are again taken into account, the number of bursts $z_{B}\left(a\right)$ is obtained.

The frequencies are normalized with respect to the non-zero elements, resulting in the burst weight–length density function bm(ℓ,g). To complete the numerical example, the length–weight distribution bm(ℓ,g) is analyzed for a=3.

Given a known density function, it is then checked whether the generated bit sequence agrees with the ideal characteristic given in (5).

As a quality metric for assessing the approximation between the obtained gap-distribution function $u_{m}\left(k\right)$ and a reference distribution u(k), the mean square error

(26) $$E_{\min}=\sum_{k=0}^{K_{\max}-1}{\left|u\left(k\right)-u_{m}\left(k\right)\right|}^{2}\;$$

is used and minimized [33]. The parameter $K_{\max}$ denotes the maximum gap length incorporated into the analysis and optimization (see [16])

Table 6 shows the optimal parameters for the considered distributions and the associated errors from the approximation. The best results were obtained for the ideal distribution (5), since for α=1, the Weibull distribution reduces to the exponential distribution. This also holds for the Wilhelm distribution at α=1, highlighting that the ideal characteristic corresponds to exponential decay.

**Figure 13:**
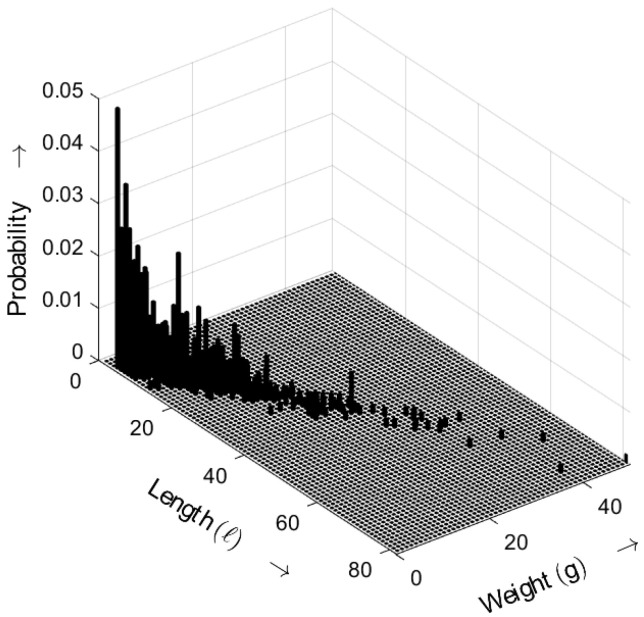
Burst-related length–weight distribution bm(ℓ,g) of the ideal random bit sequence for a=3. Occurrence probabilities of bursts as a function of length *ℓ* and weight *g*.

Figure 13 shows the length–weight distribution obtained for the ideal random sequence (i.e., a random sequence with an exponential gap distribution) as defined by (5), with the distance parameter a=3. The three-dimensional plot shows the probability of bursts as a function of both their length *ℓ* and weight *g*.

Designating a non-zero element in the random sequence as *L*, the probability that two successive elements of the random sequence are non-zero is given by

(27) P(LL)=P(L)·P(L|L),

where $P\left(L\right)=p_{e}$ is the probability that an arbitrary element of the sequence is non-zero and P(L|L) is the conditional probability that a non-zero element is immediately followed (i.e., with gap length k=0) by another non-zero element. In this case, the gap has length k=0 occurs, and

(28) P(L|L)=v(0)=1−u(1)

holds, since v(0) represents exactly the probability that a non-zero element is immediately followed by another non-zero element.

To evaluate whether the random variables are independent and identically distributed and follow a discrete uniform distribution, in other words, whether the IID-DU assumption is satisfied, it is important to determine v(0)—the probability that one non-zero element immediately follows another in the random sequence. If the IID-DU assumption holds, then v(0)=0.5.

This probability can be computed by analyzing the gap distribution using (3). For k=0,

(29) v(0)=u(0)−u(1)

and for u(0)=1, it follows that

(30) v(0)=1−u(1).

Here, u(1) denotes the probability that a gap of length k≥1 occurs, corresponding to the number of bursts defined using the distance parameter a=1.

In the sequence under investigation, there are $z_{f}=5042$ non-zero elements and 2481 gaps with k≥1 (corresponding to the number of bursts for a=1). This yields u(1)=2481/5042 and v(0)=1−2481/5042=1−0.4921≈0.5079. Therefore, the IID-DU requirement is approximately satisfied.

Furthermore, the gap-distribution function u(k) can be easily determined by calculating the number of bursts with a given distance parameter *a*, according to

(31) $$u\left(k\right)=\frac{\mathrm{number}\;\mathrm{of}\;\mathrm{bursts}\;\mathrm{with}\;\;a=k}{z_{f}}\;.$$

Table 7 shows the gap distribution determined from the burst distribution for the exemplary random sequence using (31). The value v(0)=0.508, obtained by calculating the difference between u(0) and u(1), confirms that the IID-DU assumption is satisfied.

If the random sequence exhibits good random behavior, the bursts are more widely distributed in terms of the non-zero elements they contain. In contrast, when the number of bursts is limited to only a few combinations, the sequence is not very random and should be regarded as a poor random sequence.

So far, the analysis has focused on the distance parameter a=3. In order to broaden the scope of the results, another numerical value for the distance parameter is investigated (a=5).

**Figure 14:**
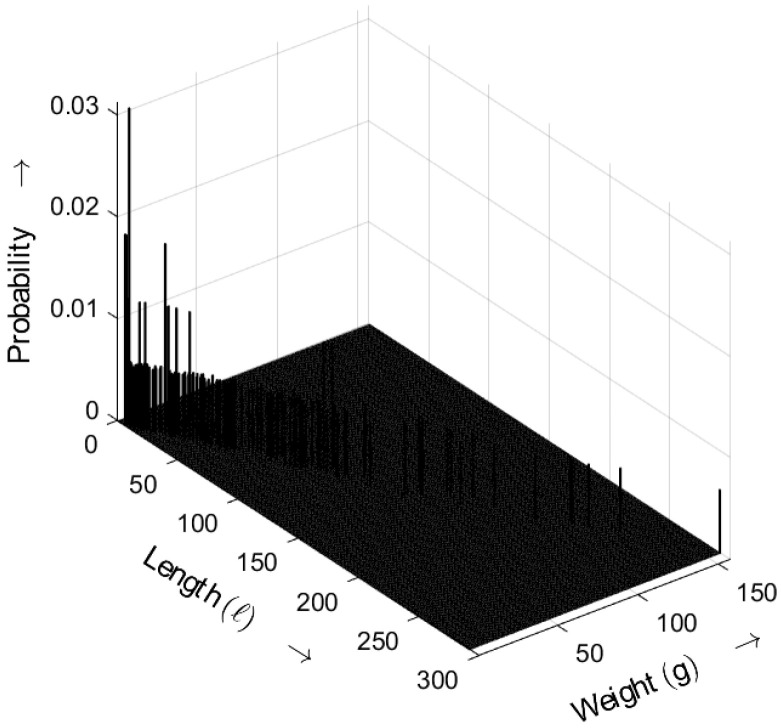
Burst-related length–weight distribution bm(ℓ,g) of the ideal random bit sequence for a=5. Occurrence probabilities of bursts as a function of length *ℓ* and weight *g*.

Figure 14 shows the length–weight distribution obtained for an ideal random sequence, derived from (5) with a=5. The three-dimensional plot shows the probability of bursts as a function of both their length *ℓ* and weight *g*. Compared with the results for a=3, the probabilities for a=5 shift toward smaller values.

## 4. Sequence Generation and Practical Verification

In this section, several approaches for generating random sequences are analyzed in terms of their characteristics and their quality with respect to randomness. To generate the length–weight distribution, a computer algebra system or a programming environment such as Matlab R2024a or Python Version 3.12 can be used in order to implement and automate the necessary procedures and calculations.

### 4.1. *m*-Sequences

A commonly used approach for generating random sequences involves maximum-length sequences, or *m*-sequences [34]. They can be easily generated in practice using linear feedback shift registers. The term “maximum” refers to the maximal period of the sequence generated by a linear feedback shift register (LFSR). For an *m*-sequence, the period has a length of $2^{n}-1$, where *n* is the length of the register. This maximal period makes them particularly useful for applications requiring long, uniformly distributed pseudorandom sequences. However, it should be noted that the generated random sequences are periodic, and the resulting burst patterns are limited to a small number of constellations.

The maximum-length sequence (*m*-sequence) analyzed here was generated using the primitive polynomial $P\left(x\right)=x^{5}+x^{2}+1$ and an LFSR approach [35], resulting in a sequence length of 10,000 bits.

**Figure 15:**
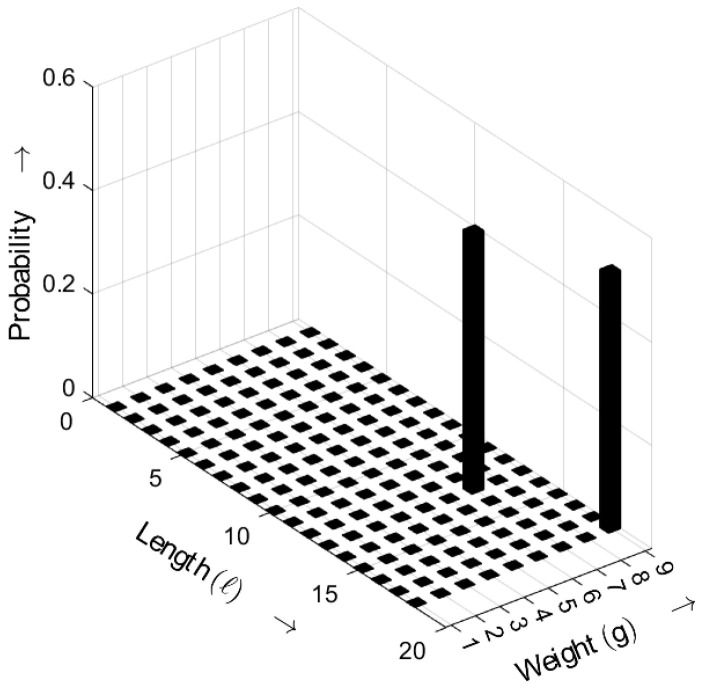
Burst-related length–weight distribution bm(ℓ,g) of an *m*-sequence for a=3. Occurrence probabilities of bursts as a function of length *ℓ* and weight *g*.

In Figure 15, the length–weight distribution of the *m*-sequence is depicted. Only two length–weight combinations exhibit non-zero probabilities, highlighting the periodic nature of LFSR-generated sequences [18].

Table 8 shows the optimal parameters for the considered distributions and the associated errors when approximating the gap distribution.

The results are somewhat similar to those of the ideal distribution (5) since the Weibull distribution reduces to the exponential distribution for α=1. This same holds for the Wilhelm distribution.

Since v(0)=0.5, the IID-DU condition is satisfied.

### 4.2. Non-*m*-Sequences

A non-*m*-sequence is a random sequence that does not exhibit the specific characteristics of an *m*-sequence [36]. The non-*m*-sequence analyzed here was generated using the polynomial $P\left(x\right)=x^{5}+x^{4}+x^{3}+1$, resulting in a sequence length of 10,000 bits. The generation process followed a similar approach to that used for the *m*-sequence, i.e., an LFSR approach.

**Figure 16:**
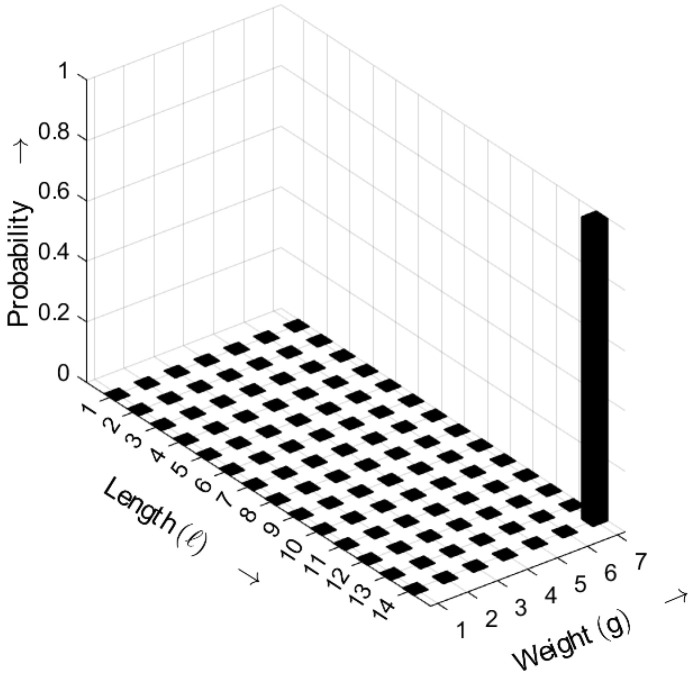
Burst-related length–weight distribution bm(ℓ,g) of a non-*m*-sequence for a=3. Occurrence probabilities of bursts as a function of length *ℓ* and weight *g*.

In Figure 16, the length–weight distribution of the non-*m*-sequence is shown. In this case, only one length–weight combination exhibits a non-zero probability; specifically, the pattern with ℓ=14 and g=7 occurs consistently. The exact output depends on the chosen polynomial, the initial state of the registers, and the distance parameter *a*.

Table 9 shows that the Wilhelm distribution provides a good approximation for the non-*m*-sequence but with larger deviations from the ideal exponential distribution, since α≠1. With v(0)=0.571, the IID-DU condition is not satisfied.

### 4.3. Bit-Sequence Generation from a Text

A text excerpt from the chapter titled “The Period” from the book *A Tale of Two Cities* by Charles Dickens [37] was used to generate a sequence of zeros and ones. The conversion process involved encoding each character of the text into its corresponding ASCII value and then converting that ASCII value into an 8-bit binary representation. However, some special characters, like the en dash (‘–’), were represented using extended ASCII or Unicode values. For instance, the en dash has a Unicode value of 8211, which requires more than the standard 8 bits for its binary representation, resulting in a 14-bit binary sequence: “10000000010011”. The concatenation of these binary strings resulted in a bit sequence with a total length of 7566 bits, representing the entire text of that chapter. For example, the word “and” was represented as three ASCII values: ‘a’ (97), ‘n’ (110), and ‘d’ (100). These values were then converted to their binary equivalents: 97 became “01100001”, 110 became “01101110”, and 100 became “01100100”. Concatenating these binary strings produced the final bitstream “011000010110111001100100” for “and”. This process was applied to the entire text, creating a binary sequence.

**Figure 17:**
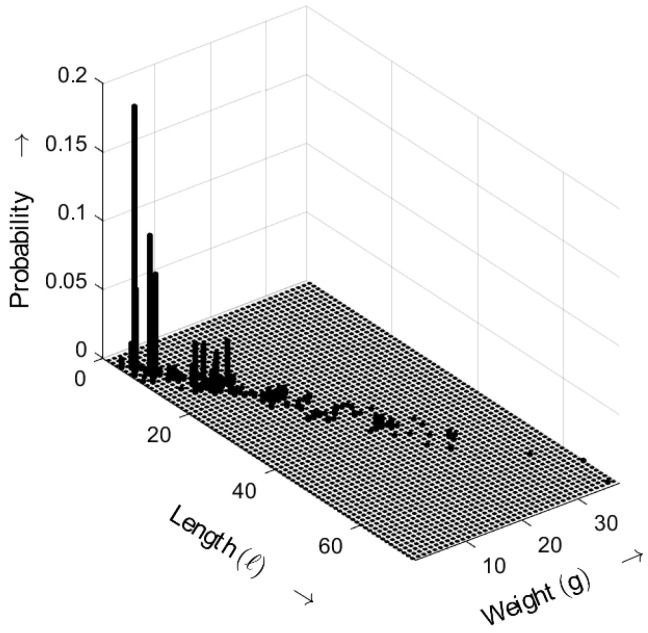
Burst-related length–weight distribution bm(ℓ,g) of a random sequence generated from a text [37] with a=3. Occurrence probabilities of bursts as a function of length *ℓ* and weight *g*.

In Figure 17, the length–weight distribution of the sequence generated from the given text [37] is shown. The length–weight combinations are concentrated on a diagonal characterized by increasing lengths and weights. With rising *ℓ* and *g*, the probabilities descend rapidly, following the ideal characteristic and exhibiting an exponential distribution.

Table 10 shows that in cases where the random sequence is produced from a literature text, a relatively good approximation to the ideal distribution is obtained, even though the residual error is rather large.

With v(0)=0.443, the IID-DU condition is again not satisfied.

### 4.4. Bit Sequence Generated Using an Online Tool

A bit sequence of length 10,000 was generated using an online tool available at https://www.browserling.com/tools/random-bin (accessed on 5 March 2025). The website offers a feature specifically designed for generating random binary sequences, which was utilized in this case. By selecting the option to create a binary sequence of the desired length, the tool randomly produced a sequence of 0s and 1s.

**Figure 18:**
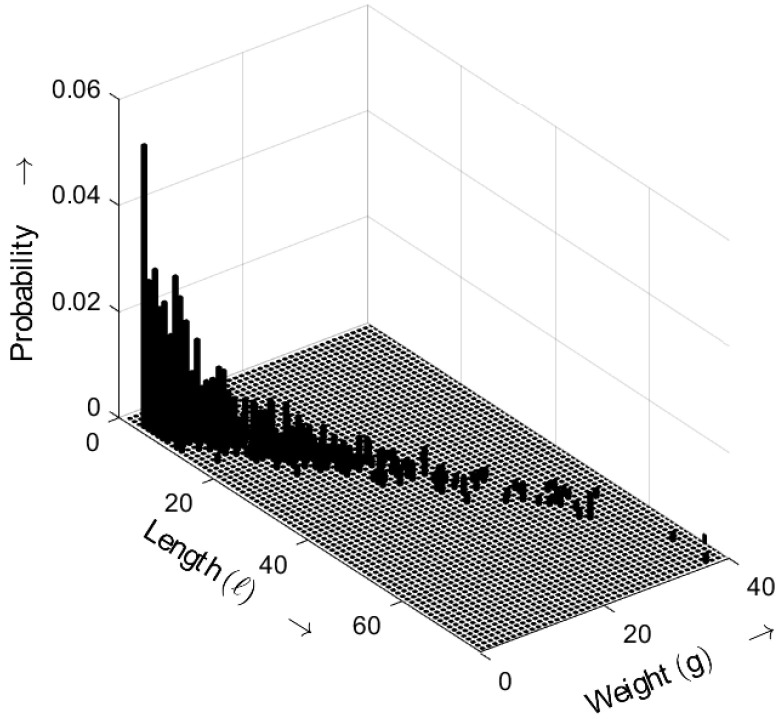
Burst-related length–weight distribution bm(ℓ,g) of a random sequence obtained using an online tool for a=3. Occurrence probabilities of bursts as a function of length *ℓ* and weight *g*.

In Figure 18, the length–weight distribution of the sequence obtained from the online tool is depicted. The length–weight combinations again follow the desired exponential characteristic.

Table 11 shows that when using the online tool for sequence generation, a good approximation of the gap distribution to the ideal distribution can be achieved, with a relatively small residual error.

The value v(0)=0.51 confirms that the IID-DU condition is satisfied.

### 4.5. NIST Test Results

For assessing randomness within binary sequences, the NIST test suite [4] plays an important role. The monobit test within the suite [4] is frequently employed as an initial statistical tool. It evaluates the balance between zeros and ones, thereby offering a preliminary indication of whether a sequence may be regarded as independent and identically distributed (IID). However, while the test confirms the equal distribution of elements, it does not account for their structural arrangement or mutual independence. Therefore, additional parameters, such as the gap distribution v(k), are necessary to gain a more comprehensive understanding of the underlying randomness characteristics.

While the elements of the bit sequence $s_{\ell }\in \left\{0,1\right\}$ are represented as binary numbers, the frequency (monobit) test requires bipolar values, which can be generated using the mapping rule ${\tilde{s}}_{\ell }=2\cdot s_{\ell }-1$ with ${\tilde{s}}_{\ell }\in \left\{-1,1\right\}$. By taking the sum of the random elements ${\tilde{s}}_{\ell }$ (with *n* elements) into account, a new random variable ${\tilde{S}}_{n}$ can be created as

(32) $${\tilde{S}}_{n}={\tilde{s}}_{1}+{\tilde{s}}_{2}+\ldots +{\tilde{s}}_{n}.$$

According to the *Moivre*–*Laplace* central limit theorem [38], the random variable ${\tilde{S}}_{n}$ divided by $\sqrt{n}$ can be approximated by a standard normal distribution, a normal distribution with zero mean and unit variance. For such a random sequence, the plus and minus ones compensate each other in the random variable ${\tilde{S}}_{n}$ -, and the value ${\tilde{S}}_{n}$ tends to be zero.

The quantity $s_{\mathrm{obs}}$ is obtained as the absolute value of the sum ${\tilde{S}}_{n}$ divided by $\sqrt{n}$ according to

(33) $$s_{\mathrm{obs}}=\frac{\left|{\tilde{S}}_{n}\right|}{\sqrt{n}}.$$

The value $s_{\mathrm{obs}}$ can now be analyzed as an indicator of the level of randomness. In cases where the bit sequence $s_{\ell }$ has too many ones or zeroes—compared to a uniform distribution of zeroes and ones—$s_{\mathrm{obs}}$ will be greater than zero. According to [4], the calculated *p*-value is obtained as

(34) $$p\text{-}\mathrm{value}=\mathrm{erfc}\left(\frac{s_{\mathrm{obs}}}{\sqrt{2}}\right).$$

A small *p*-value or large $s_{\mathrm{obs}}$ (based on the monotonicity property of the complementary error function erfc(x)) denotes a rather non-random behavior. Here, a *p*-value smaller than $10^{-2}$ or $s_{\mathrm{obs}}>2.59$ is seen as an indicator that either too many ones (leading to a large positive value of ${\tilde{S}}_{n}$) or too many zeroes (leading to a large negative value of ${\tilde{S}}_{n}$) are in the sequence.

The monobit test evaluates the ratio of zeros to ones in a binary sequence but does not take into account their positional distribution within the bitstream. In accordance with the assumption of independent and identically distributed (IID) elements, the monobit test exclusively verifies whether zeros and ones are identically distributed. This condition is satisfied when the empirical probability of each element, $p_{e}$, equals 0.5, or equivalently, when the observed statistic $s_{\mathrm{obs}}=0$ (checking for identical distribution). However, the monobit test does not establish a connection to the gap-distribution function v(k), particularly with respect to v(0), where v(0)=0.5 would indicate statistical independence (checking for independence). A result of $p_{e}=0.5$ merely indicates that the likelihood of encountering a zero or one is equal, each occurring with a probability of 50%. As a consequence, the monobit test may classify sequences as preliminarily random, even if they satisfy the condition $p_{e}=0.5$, while simultaneously violating v(0)=0.5. This highlights a critical limitation of the test: sequences can appear balanced in terms of frequency yet still exhibit non-random structural dependencies.

The results obtained from the monobit test are presented in Table 12 and compared against the corresponding values of the gap function v(0).

The findings demonstrate that the ideal sequence satisfies the IID (independent and identically distributed) condition, as indicated by a *p*-value greater than 0.01 and a gap value of v(0)≈0.5. In contrast, the analyzed *m*-sequence of length N=10,000 can only be regarded as nearly random. This is due to its failure to meet the required *p*-value threshold (here, *p*-value = 0.001), which implies a violation of the IID assumption, as evidenced by a slight imbalance in element frequency, with more ones than zeros (i.e., $p_{e}=0.516$). The examined non-*m*-sequence represents a special case. While it passes the monobit test and could therefore be preliminarily classified as random, its gap value of v(0)=0.57 (checking for independence) reveals a lack of statistical independence. This is particularly noteworthy, as the sequence contains an equal number of ones and zeros, suggesting balance but not independence. The *bit sequence generated from a text* fails both conditions—p-value≥0.01 and v(0)≈0.5—and must therefore be classified as non-random. Conversely, the *bit sequence generated using an online tool* satisfies both conditions and can be considered random. In summary, the monobit test provides an initial indication of compliance with the IID assumption by verifying the balance between the number of zeros and ones. However, it alone is insufficient for a comprehensive randomness assessment, particularly with respect to structural independence within the sequence.

Ultimately, while the monobit test offers valuable insights into the distributional balance of binary sequences, it must be complemented by further analytical methods—such as gap-based evaluations—to adequately assess both distribution and independence. This combined approach enables a more robust and reliable characterization of randomness, particularly in contexts requiring high statistical rigor, such as cryptographic systems or stochastic modeling.

### 4.6. Comparison of the Results

In this section, various approaches for generating random sequences are reviewed with regard to their quality.

To analyze the burst matrix, as shown earlier, the independence of the elements and their distribution requires a wider distribution of the non-zero elements within the length–weight matrix, rather than only a few non-zero elements in that matrix, as in the case of an *m*-sequence or a non-*m*-sequence.

Although *m*-sequences satisfy the IID-DU condition, the restriction to a few non-zero elements in the burst matrix (see Figure 15) is a security risk in cryptographic applications, since an inverse operation does not seem impossible (e.g., concluding the generator polynomial from the burst matrix). Few non-zero elements in the burst matrix also increase the risk of a violation of the IID-DU property, as shown in the analysis of the non-*m*-sequence (see Figure 16).

In addition, common distribution functions, such as Rayleigh, Weibull, or exponential distributions, were analyzed with regard to their suitability for describing the distribution of the gaps. The ideal distribution function with regard to the IID-DU assumption can be described by a single parameter $p_{e}$ together with the additional condition $p_{e}=0.5$ and $v\left(0\right)=p_{e}$ and leads to an exponential distribution function.

**Figure 19:**
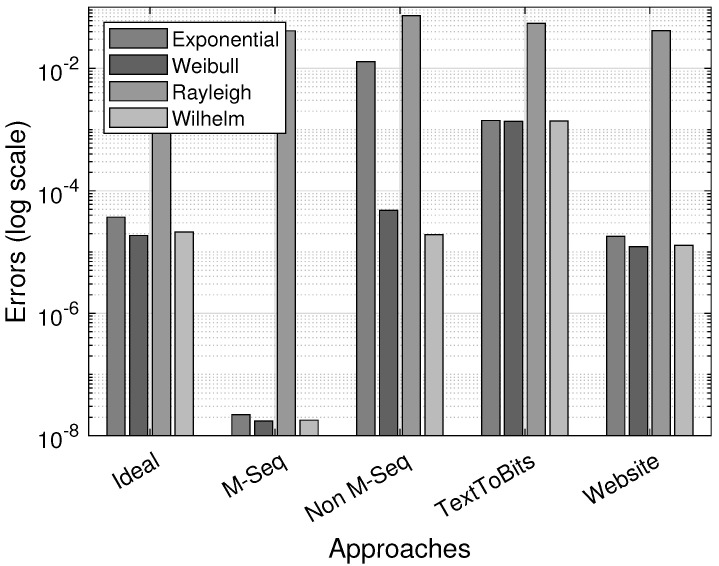
Error comparison across the different bitstream-generation approaches.

Figure 19 shows the errors when estimating the optimized parameters of the underlying gap-distribution function. The smallest estimation errors for sequences with the IID-DU assumption can be obtained with distribution functions with one parameter. Furthermore, with α close to 1, the Weibull distribution, as well as the Wilhelm distribution, is close to the exponential one.

## 5. Post-Quantum Cryptographic Approaches

Randomness plays a pivotal role in various cryptographic processes, often resulting in sequences that are more random than deterministic. In post-quantum cryptography (PQC), this aspect of randomness is particularly significant. Two key cryptographic schemes from PQC, CRYSTALS-Kyber and CRYSTALS-Dilithium, are rooted in lattice-based problems, which rely heavily on randomness for their security. One foundational problem in this context is the Learning with Errors (LWE) problem. As Oded Regev discussed in [39], LWE forms the backbone of many lattice-based cryptographic schemes, including those found in the CRYSTALS suite. The LWE problem is computationally hard to solve, even for quantum computers. It involves solving a system of linear equations that are perturbed by small random errors, or noise. This noise significantly complicates the problem, especially in high-dimensional spaces, making it resistant to both classical and quantum attacks. The security of CRYSTALS-Kyber (key exchange) and CRYSTALS-Dilithium (digital signatures) is directly tied to the intractability of LWE, thus ensuring their resilience in the post-quantum era.

The generic approach presented in this article is applicable to all cryptographic schemes and is particularly relevant in light of the threats quantum computers pose to classical asymmetric algorithms such as RSA (Rivest–Shamir–Adleman) and ECC (Elliptic Curve Cryptography). In contrast, symmetric algorithms like AES and ChaCha20 are believed to be quantum-resistant. From an evaluative standpoint, our test lab focuses specifically on post-quantum cryptography (PQC) candidates [40,41,42].

The most significant challenge in the implementation of post-quantum cryptography (PQC) lies in the distribution of cryptographic keys and algorithms that are provably secure against quantum attacks [43]. From a PQC perspective, in [44], Farooq et al. discussed the performance and resilience optimization of public-key encryption (PKE), key-establishment mechanisms (KEMs), and digital signature algorithms (DSAs), which are fundamental cryptographic protocols for securing communication over insecure or untrusted networks and ensuring both the confidentiality and integrity of exchanged data. As Bernstein et al. described in [45], unlike common cryptographic methods such as RSA, ECC, and ECDSA (Elliptic Curve Digital Signature Algorithm), cryptographic hash algorithms such as SHA (Secure Hash Algorithm) are vulnerable to quantum computers and not designed to remain secure against attacks by quantum computers or quantum algorithms. With the practical use of Shor’s polynomial-time algorithm [46], the computational hardness problems, such as prime factorization and the discrete logarithm problem, are effectively solved. Within the work of Hasija et al. [47], the most promising PQC candidate is the lattice-based approach with the well-studied CRYSTALS-Kyber for key-encapsulation and key-exchange and CRYSTALS-Dilithium for digital signature. The security for both schemes is based on the mathematical problem of the lattice problem, which is considered mathematically hard to solve, even for quantum computers. This mathematical foundation provides a high level of security. Both PQC algorithms are candidates for NIST selection [48] for standardized post-quantum cryptography algorithms [49].

CRYSTALS-Kyber and Dilithium serve different cryptographic purposes, but their underlying mathematical schemes provide a fundamental point of similarity and define the relationship between Kyber and Dilithium.

### 5.1. CRYSTALS-Kyber

The first algorithm that is evaluated is the Kyber algorithm contained in CRYSTALS. As a central one-way function, the LWE problem in a modular lattice is used. CRYSTALS-Kyber is a key-exchange protocol that enables two parties to exchange a shared secret key over an insecure communication channel. This key can then be used by symmetric encryption methods to keep communication confidential. CRYSTALS-Kyber combines the benefits of public-key and symmetric-key cryptography and handles key exchange rather than directly encrypting or decrypting data. In practical systems, the shared secret generated via CRYSTALS-Kyber is used between two parties and is then used as a key for a symmetric encryption algorithm like AES (Advanced Encryption Standard) to encrypt and decrypt the actual data. CRYSTALS-Kyber handles the secure exchange of the symmetric AES key, which is used for the actual encryption of data. The security of Kyber relies on introducing a small amount of noise (error) in specific computations, which makes reversing these operations computationally infeasible without access to the private key. This noise is generally sampled from a discrete Gaussian distribution or other similar error distributions. The cryptographic strength of CRYSTALS-Kyber is based on the difficulty of solving the Ring-LWE problem, a lattice-based problem considered resistant to quantum attacks, thereby providing strong post-quantum security.

The Kyber algorithm, as implemented in the current Open Quantum Safe (OQS) library, supports three security levels: kyber-512, kyber-768, and kyber-1024. Each of these variants corresponds to a specific lattice dimension, which directly influences the security properties of the cryptographic scheme. In the context of Kyber, the lattice dimension determines the size of the vectors used in key-generation, encapsulation, and decapsulation operations. Lower-dimensional variants, such as kyber-512 and kyber-768, offer reduced post-quantum security levels of approximately 128 bits and 192 bits, respectively. In contrast, the higher-dimensional kyber-1024 provides enhanced security with its larger lattice, achieving around 256-bit post-quantum security, making it suitable for applications requiring stronger cryptographic guarantees. For the test environment, the OQS library OQS_KEM_alg_kyber_1024 with the following parameters was used: the algorithm variant Kyber1024 from the type KEM, defined as Indistinguishability under Adaptive Chosen-Ciphertext Attack (IND-CCA) at security level 2, with a claimed NIST level of 5. The approximate output length for OQS_KEM_alg_kyber_1024 components in bytes was private/secret key: 3168; public key: 1568; ciphertext: 1568; and shared secret: 32. Upon successful compilation, the binary file was executed 1000 times via a shell script. During each iteration, 1000 unique key pairs—comprising both private/secret and public keys—were generated, along with the corresponding ciphertexts and shared secrets. These outputs were subsequently encoded into bitstreams and written to 1000 distinct files for further processing.

**Figure 20:**
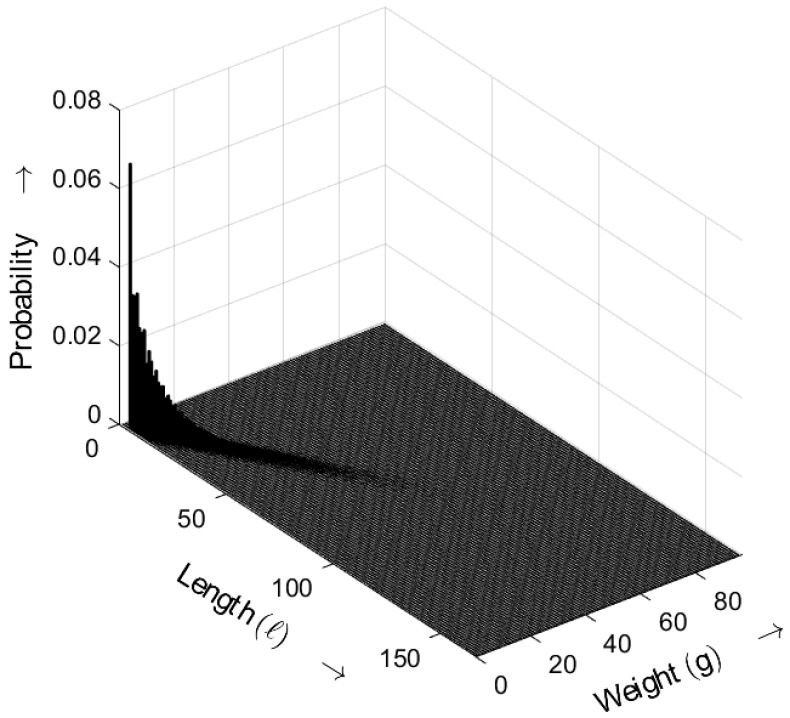
Burst-related length–weight distribution bm(ℓ,g) of random sequence generated by CRYSTALS-Kyber for a=3. Occurrence probabilities of bursts as a function of length *ℓ* and weight *g*.

In Figure 20, the length–weight distribution of the ciphertext is shown, which was generated by applying the CRYSTALS-Kyber key-exchange protocol. The distribution shows the expected randomness in the ciphertext without anomalies. The probabilities decrease with increasing length and weight.

Table 13 shows a good approximation to the ideal distribution with medium residual error.

The value v(0)=0.464 indicates that the IID-DU condition was, on average, nearly satisfied.

### 5.2. CRYSTALS-Dilithium

CRYSTALS-Dilithium is a digital signature scheme that is based on the *Fiat–Shamir with Aborts* approach. In essence, the traditional Fiat–Shamir heuristic [50] allows for aborting the signing process under certain conditions. Thereby, the robustness and the security of the Fiat–Shamir heuristic in cryptographic applications are ensured, which is related to Vadim Lyubashevsky’s research [51], and efficiency is improved through the combination of Ring-LWE and Ring-SIS [52]. Reducing the signature size improves the scheme [53,54]. Basically, it is an identification scheme that has been adapted to the application of lattices. CRYSTALS-Dilithium allows for the digital signing of data or messages, ensuring that the authenticity and integrity of the message can be verified. A digital signature ensures that a message originates from a specific person or system and that the message has not been altered since it was signed. CRYSTALS-Dilithium has several variants that allow for different security levels. These correspond to lattices with an increasing number of dimensions and result in different key and signature lengths. The security of CRYSTALS-Dilithium is rooted in the computational hardness of the Module-LWE and Module-SIS problems. These lattice-based problems are believed to be resistant to even quantum attacks, providing robust post-quantum security. The following process focuses on examining the randomness of the binary file generated by the signature. The OQS library provides post-quantum digital signatures based on the CRYSTALS-Dilithium scheme. The CRYSTALS-Dilithium signature scheme operates through three main phases: key generation, signing, and verification. In this process, the generic OQS_SIG_alg_dilithium implementation generates secure key pairs, where the private key is used to create digital signatures, and the public key is used to verify them. The OQS library supports several variants of the Dilithium algorithm, including OQS_SIG_alg_dilithium_2 and OQS_SIG_alg_dilithium_3, which offer medium and high security levels. The highest level of security is provided by the OQS_SIG_alg_dilithium_5 variant. In general, a higher number indicates stronger security at the cost of increased runtime overhead. Using standard C++ libraries, a 1000-digit alphanumeric plaintext input file was created per iteration, with the content randomized, mocked, and persisted. Subsequently, a digital signature for the plaintext file was created and saved as a sig binary file type, which was later encoded into bitstreams. The Dilithium2 algorithm Dilithium2 from the type signature has a claimed NIST level of 2. The approximate output length for OQS_SIG_alg_dilithium_2 components in bytes was private/secret key: 2528; public key: 1312; and signature: 2420. These outputs were subsequently encoded into bitstreams and written to 1000 distinct files for further processing and analysis tasks.

**Figure 21:**
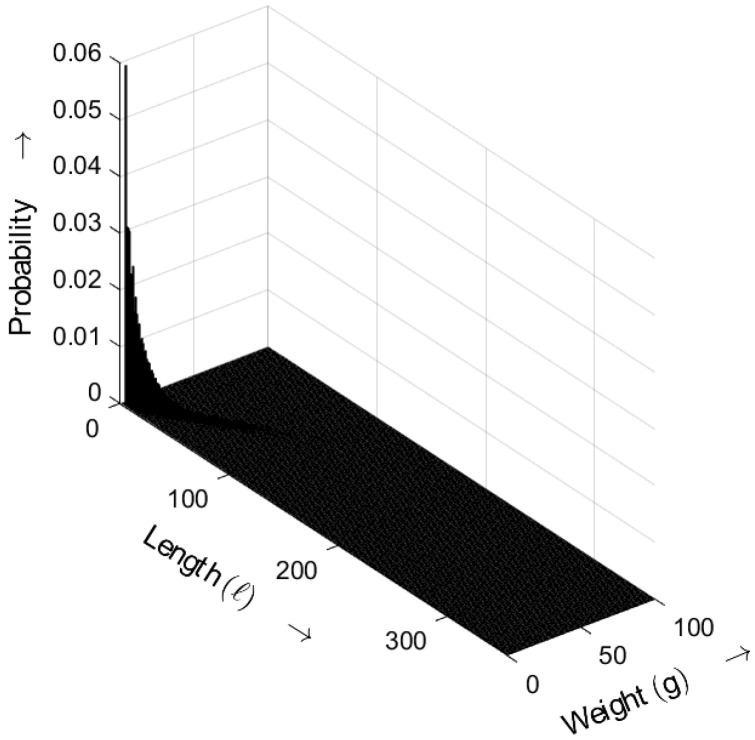
Burst-related length–weight distribution bm(ℓ,g) of random sequence generated by CRYSTALS-Dilithium2 for a=3. Occurrence probabilities of bursts as a function of length *ℓ* and weight *g*.

In Figure 21, the length–weight distribution of signatures (averaged) is shown, which were generated by applying the CRYSTALS-Dilithium2 signature algorithm. CRYSTALS-Dilithium2 is one of several variants of the CRYSTALS-Dilithium signature algorithm suite. The distribution shows the expected randomness in the signatures without anomalies. The probabilities decrease with increasing length and weight.

Table 14 confirms a relatively good approximation to the ideal distribution with small residual error.

The value v(0)=0.499 indicates that the IID-DU condition was satisfied.

### 5.3. Comparison of Cryptographic Approaches

**Figure 22:**
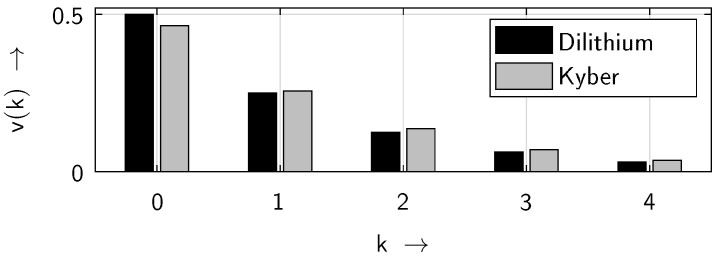
Gap probability density functions v(k) for Dilithium and Kyber.

In Figure 22, the probability density functions of the gap distributions for both approaches, Dilithium and Kyber, are shown. It can be seen that Dilithium ensures a higher level of randomness than Kyber.

While CRYSTALS-Dilithium exhibits excellent randomness with a value of v(0)=0.499—very close to the ideal value of v(0)=0.5—the level of randomness of CRYSTALS-Kyber is lower, exhibiting a value of only v(0)=0.464.

## 6. Conclusions

The distribution of the lengths and weights of bursts is a characteristic statistical property of randomized bit sequences and can therefore be used to analyze their randomness. In this work, a new approach for analyzing randomness in binary sequences has been developed and tested on several computer-generated binary random sequences. The parameter v(0), representing the probability that a “1” is immediately followed by another “1”, serves as a diagnostic for randomness—ideally equating to 0.5 for independent and identically distributed (IID) sequences with a discrete two-point uniform distribution (DU)—yet deviations from this benchmark can arise due to statistical fluctuations in short sequences or structural correlations inherent in real-world cryptographic systems. Thus, while v(0) is a valuable and computationally efficient metric, it should not be interpreted in isolation but rather complemented by additional statistical tests (e.g., entropy estimation, run-length analysis, or model fitting) to distinguish between genuine anomalies and benign design-related deviations. While well-established approaches focus on frequency or run-length tests, the newly developed technique emphasizes the length–weight distribution of non-zero elements in random bit sequences. The gap-based approach allows for estimating corresponding statistical parameters such as the gap-density function, which is a strong indicator of the IID-DU condition. The observed deviations, especially in the short-gap region (e.g., v(0)), highlight potential vulnerabilities in randomness quality [55,56]. Although the current results do not claim immediate cryptanalytic exploitation, they lay the groundwork for future studies that could translate such deviations into entropy loss metrics or distinguisher-based attack models. As cryptographic systems move toward post-quantum resilience, incorporating such structural analyses into the validation pipeline will be critical for ensuring robustness and trustworthiness. For testing purposes, conventional schemes such as *m*-sequences and non-*m*-sequences have been analyzed, and cryptographic schemes such as Kyber and Dilithium have been studied. While Kyber and Dilithium are designed to produce statistically uniform outputs [57], the claim that Kyber exhibits lower randomness remains speculative without rigorous empirical validation. The current literature lacks direct comparative analyses of their randomness characteristics. Therefore, such assertions should be approached cautiously and warrant further statistical investigation. As the present study focuses on establishing a novel methodological framework, a detailed comparative evaluation is reserved for future work. The results underline that the gap-based burst approach is suitable for analyzing randomness in binary sequences.

## 7. Future Work

In future work, the gap-based burst methodology will be further developed to complement and enhance traditional test suites like NIST, TESTU01, and DieHard by jointly assessing distribution and independence through the analysis of gap patterns between significant bits. To evaluate the observed differences in randomness, both the Kolmogorov–Smirnov test and the two-sample *t*-test will be applied to the gap-density functions derived from multiple independent ciphertexts. The results are expected to reveal a statistically significant difference, with Kyber showing a higher concentration of short-gap frequencies. This suggests measurable structural variations in bit-level entropy between the two PQC schemes. This expanded approach aims to more effectively detect subtle correlations and structural anomalies in binary sequences—an essential capability for cryptographic evaluations. While the current study demonstrates the potential of burst-based and gap-distribution methods for detecting structural irregularities in binary sequences, further research is required to assess their practical cryptographic relevance. One promising direction is the quantitative estimation of entropy loss associated with observed deviations from ideal IID-DU distributions. This could involve developing attacker models such as statistical distinguishers or entropy estimators to simulate how these structural weaknesses might be exploited in realistic cryptanalytic scenarios. Additionally, expanding the methodology to include adaptive detection mechanisms or integration into post-quantum cryptographic benchmarking suites would further validate its utility across broader algorithm classes and implementation contexts. The methodology’s reliance solely on raw output bits enables its application across a wide range of post-quantum cryptographic (PQC) schemes, including SIKE (Supersingular Isogeny Key Encapsulation), Rainbow, and Falcon (Fast Fourier Lattice-Based Compact Signatures over NTRU). Upcoming work will focus on refining its use as a portable, scheme-agnostic tool for evaluating the randomness quality of cryptographic outputs, thereby strengthening its utility in the evolving landscape of PQC standardization [58,59,60].

## Figures and Tables

**Table 1:** Suitable probability distributions.

Type	Probability Density	Distribution u(k)
Exponential	$\beta_{e}\;e^{-\beta_{e}k}$	$e^{-\beta_{e}k}$
Weibull	$\beta_{w}{\left(-\beta_{w}k\right)}^{\alpha_{w}-1}\;e^{-{\left(\beta_{w}k\right)}^{\alpha_{w}}}$	$e^{-{\left(\beta_{w}k\right)}^{\alpha_{w}}}$
Rayleigh	$\beta_{r}^{2}\;k\;e^{-\beta_{r}^{2}\frac{k^{2}}{2}}$	$e^{-\beta_{r}^{2}\frac{k^{2}}{2}}$

**Table 2:** Number of measured bursts $z_{B}\left(a\right)$ in the analyzed sequence.

*a*	Theory	Simulation
2	1260	1215
3	630	624
5	158	160

**Table 3:** Probability P(g) for different values of the parameter *a* in the analyzed sequence (the number of bursts at the given weight is shown in parentheses).

*a*	P(g=1)	P(g=2)	P(g=3)
2	0.239 (289)	0.172 (209)	0.137 (167)
3	0.112 (70)	0.112 (70)	0.093 (58)

**Table 4:** Theoretical values for the probability P(g) for different values of the parameter *a* derived from (4).

*a*	P(g=1)	P(g=2)	P(g=3)
2	0.250	0.188	0.141
3	0.125	0.109	0.096

**Table 5:** Burst-related length–weight distribution for the ideal random bit sequence with the distance parameter a=3.

Length *ℓ*	Weight *g*						∑ℓ
	1	2	3	4	5–30	31–50	
4	31	0	0	0	0	0	31
5	17	22	0	0	0	0	39
6	11	17	13	0	0	0	41
7	7	13	15	6	0	0	41
8–40	4	18	30	40	358	0	450
41–81	0	0	0	0	11	11	22
∑g	70	70	58	46	369	11	624

**Table 6:** Optimal parameters for the distribution functions.

Distribution	α	β	$E_{\min}$
Exponential	–	0.706	$3.71\times 10^{-5}$
Weibull	0.986	0.709	$1.86\times 10^{-5}$
Rayleigh	–	1.0011	$4.20\times 10^{-2}$
Wilhelm	0.984	0.689	$2.13\times 10^{-5}$

**Table 7:** Observed parameters for the gap-distribution function ($z_{f}=5042$).

*k*	$z_{B}\left(a=k\right)$	u(k)
0	5042	1.00
1	2481	0.49
2	1215	0.24
3	624	0.13
4	305	0.06
5	160	0.03

**Table 8:** Obtained estimated parameters of the distribution functions using an *m*-sequence.

Distribution	α	β	$E_{\min}$
Exponential	–	0.694	$2.2\times 10^{-8}$
Weibull	0.999	0.694	$1.74\times 10^{-8}$
Rayleigh	–	0.978	$4.11\times 10^{-2}$
Wilhelm	0.999	0.693	$1.79\times 10^{-8}$

**Table 9:** Obtained estimated parameters of the distribution functions using a non-*m*-sequence.

Distribution	α	β	$E_{\min}$
Exponential	–	0.688	$1.28\times 10^{-2}$
Weibull	0.596	0.748	$4.82\times 10^{-5}$
Rayleigh	–	1.075	$7.28\times 10^{-2}$
Wilhelm	0.620	0.221	$1.92\times 10^{-5}$

**Table 10:** Obtained estimated parameters of the distribution functions converting text into a bit sequence.

Distribution	α	β	$E_{\min}$
Exponential	–	0.588	$1.41\times 10^{-3}$
Weibull	0.982	0.590	$1.36\times 10^{-3}$
Rayleigh	–	0.830	$5.43\times 10^{-2}$
Wilhelm	0.986	0.575	$1.38\times 10^{-3}$

**Table 11:** Obtained estimated parameters of the distribution functions using the online tool for bit-sequence generation.

Distribution	α	β	$E_{\min}$
Exponential	–	0.709	$1.80\times 10^{-5}$
Weibull	0.992	0.711	$1.22\times 10^{-5}$
Rayleigh	–	1.004	$4.12\times 10^{-2}$
Wilhelm	0.991	0.699	$1.29\times 10^{-5}$

**Table 12:** Obtained *p*-values for different bitstream-generation approaches.

Type	*p*-Value	$s_{\mathrm{obs}}$	v(0)
Ideal sequence	0.40	0.84	0.508
*m*-sequence	0.001	3.28	0.5
Non-*m*-sequence	1	0	0.57
Bit sequence generated from a text	$1.6\cdot 10^{-20}$	9.29	0.44
Bit sequence generated using an online tool	0.13	1.5	0.51

**Table 13:** Obtained estimated parameters of the distribution functions using CRYSTALS-Kyber-generated cybertext.

Distribution	α	β	$E_{\min}$
Exponential	–	0.639	$1.89\times 10^{-4}$
Weibull	1.035	0.634	$2.66\times 10^{-5}$
Rayleigh	–	0.890	$4.42\times 10^{-2}$
Wilhelm	1.042	0.679	$2.80\times 10^{-5}$

**Table 14:** Obtained estimated parameters of the distribution functions using CRYSTALS-Dilithium2-generated signatures.

Distribution	α	β	$E_{\min}$
Exponential	–	0.693	$2.69\times 10^{-5}$
Weibull	0.999	0.693	$1.38\times 10^{-5}$
Rayleigh	–	0.977	$4.25\times 10^{-2}$
Wilhelm	0.999	0.692	$1.35\times 10^{-5}$

## Data Availability

Data are contained within the article.

## Abbreviations

The following abbreviations are used in this manuscript:

AES	Advanced Encryption Standard
BOP	Bit occurrence probability
DSA	Digital signature algorithm
ECC	Elliptic Curve Cryptography
ECDSA	Elliptic Curve Digital Signature Algorithm
KEM	Key-establishment mechanism
IID	Independent and identically distributed
IP	Internet Protocol
LFSR	Linear feedback shift register
LWE	Learning with Errors
NIST	National Institute of Standards and Technology
OQS	Open Quantum Safe
PKE	Public-key encryption
PQC	Post-quantum cryptography
RSA	Rivest–Shamir–Adleman
SIKE	Supersingular Isogeny Key Encapsulation
SHA	Secure Hash Algorithm
TCP	Transmission Control Protocol
